# Effect of Soluble Factors Released from Porcine Freeze-Dried Lung Tissue (FDLT) on Modulation of Cell Growth and EMT Signature in Non-Small Cell Lung Cancer (NSCLC)—A Preliminary In Vitro Study

**DOI:** 10.3390/ijms262311743

**Published:** 2025-12-04

**Authors:** Umme Samia, Daniela Omodei, Luisa Amato, Caterina De Rosa, Rosa Camerlingo, Virna Conti, Stefano Grolli, Orlando Ferroni, Adriano Piattelli, Giovanni N. Roviello, Carminia Maria Della Corte, Viviana De Rosa, Maria Cristina Curia, Francesca Iommelli

**Affiliations:** 1 Department of Medical, Oral and Biotechnological Sciences, University “G.d’Annunzio” of Chieti-Pescara, 66100 Chieti, Italy; umme.samia@unich.it; 2 Department of Neuroscience, Imaging and Clinical Sciences, University “G.d’Annunzio” of Chieti-Pescara, 66100 Chieti, Italy; 3 Institute of Biostructures and Bioimaging, National Research Council, 80145 Naples, Italy; daniela.omodei@ibb.cnr.it (D.O.); giovanni.roviello@cnr.it (G.N.R.); viviana.derosa@ibb.cnr.it (V.D.R.); francesca.iommelli@ibb.cnr.it (F.I.); 4 Department of Precision Medicine, University of Campania Luigi Vanvitelli, 80131 Naples, Italy; luisa.amato@unicampania.it (L.A.); caterina.derosa1@unicampania.it (C.D.R.); carminiamaria.dellacorte@unicampania.it (C.M.D.C.); 5 Department of Cell Biology and Biotherapy, Istituto Nazionale Tumori-IRCCS-Fondazione G. Pascale, 80131 Naples, Italy; r.camerlingo@istitutotumori.na.it; 6 Department of Veterinary Medical Sciences, University of Parma, Via del Taglio 10, 43126 Parma, Italy; virna.conti@unipr.it (V.C.); stefano.grolli@unipr.it (S.G.); 7 Neorland Srl, Via Del Sale, 40/A, 26100 Cremona, Italy; o.ferroni@neorland.com; 8 School of Dentistry, Saint Camillus International University of Health and Medical Sciences, 00131 Rome, Italy; apiattelli51@gmail.com; 9 Facultad de Medicina, UCAM Universidad Católica San Antonio de Murcia, 30107 Murcia, Spain

**Keywords:** porcine freeze-dried lung tissue (FDLT), non-small cell lung cancer (NSCLC) cell line, epithelial–mesenchymal transition (EMT), mitochondrial membrane potential assay (MMP)

## Abstract

Lung cancer remains one of the leading causes of cancer-related mortality worldwide, with therapeutic efficacy often hindered by the development of multidrug resistance. Consequently, alternative strategies to slow down tumor progression warrant rigorous investigation. Bioactive molecules derived from tissues and organs have shown potential therapeutic properties for several diseases. We investigated the biological role of soluble bioactive factors derived from lyophilized porcine freeze-dried lung tissue (FDLT), as they may contain tumor-suppressing components involved in the progression of non-small cell lung cancer (NSCLC). NSCLC H1975 and PC9 cell lines were treated with FDLT at concentrations of 0.25 mg/mL and 0.5 mg/mL. Cell cycle analysis and mitochondrial membrane potential (MMP) assays were performed to assess cell proliferation and cell death activation. In parallel, epithelial–mesenchymal transition (EMT) markers were detected by qRT-PCR. Our findings showed that FDLT treatment reduced the viability of H1975 and PC9 cells in a dose-dependent manner, along with significant suppression of cell proliferation and colony formation. Moreover, FDLT treatment altered the cell cycle phases and determined a concomitant reduction of cyclin D1 levels as well as induction of mitochondria depolarization by suppressing MMP. Finally, qRT-PCR revealed significant downregulation of EMT-related genes vimentin and N-cadherin, along with the EMT transcription factor Twist. These findings highlight soluble FDLT-derived biomolecules as a potential tool to design alternative treatment strategies for NSCLC.

## 1. Introduction

Lung cancer is one of the leading causes of cancer-related mortality around the world [1], and it can be broadly classified into two main categories: small cell lung cancer (SCLC) and non-small cell lung cancer (NSCLC), with NSCLC accounting for 85–90% of all cases, making it the most prevalent subtype [2]. The effectiveness of conventional chemotherapeutic agents in treating lung cancer is, however, limited by their common drawbacks, which include low absorption, non-specific targeting, and the emergence of drug resistance [3]. In recent years, various efforts have been made to identify new targeted agents and treatment regimens to stop cancer progression and tumor regrowth. In this respect, several natural compounds and factors, secreted by cell populations composing tissues or organs, have shown potential benefits [4]. In particular, the complex mixture of molecules released from a specific organ, including proteins, nucleic acids, lipids, and extracellular vesicles (EVs), is defined as the secretome and is physiologically involved in regulating tissue homeostasis and regeneration [5,6,7,8,9] as well as in the physiological maintenance of organs [10]. In particular, secretome can also affect cell proliferation by regulating the tissue balance between cell cycle activation and G0 arrest [11]. Therefore, the overall effect of released factors on cell duplication depends on the specific cell source and cellular environment as well as the surrounding organ microenvironment.

In this study, we aimed to evaluate the effect of bioactive molecules derived from freeze-dried lung tissue (FDLT), prepared from healthy pig lungs processed through a carefully controlled lyophilization procedure, on NSCLC biology. The preparation method was designed to preserve the properties of components naturally present in the tissue, including nucleic acids, soluble proteins, vitamins, minerals, and essential amino acids. Overall, the present study sought to investigate the biological activity of soluble FDLT-derived biomolecules on cancer cell proliferation and viability as well as on the modulation of genes associated with cancer progression and changes in the mRNA and protein levels of epithelial–mesenchymal transition (EMT) factors [12,13].

## 2. Results

### 2.1. Circular Dichroism

**Figure 1:**
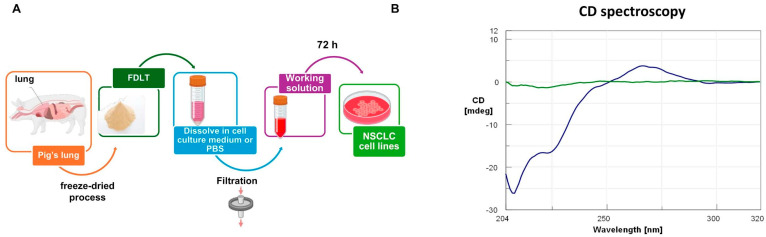
(**A**) FDLT powder dissolution in sterile aqueous buffer at a concentration of 1 mg/mL. (**B**) Circular dichroism (CD) spectrum of the soluble FDLT-derived biomolecules. The green curve represents the CD spectrum of PBS buffer, pH 7.4, alone at 25 °C. The blue curve shows the CD spectrum of the FDLT-derived biomolecular mixture in PBS buffer, pH 7.4, after subtraction of the buffer signal, highlighting the chiral optical activity of solubilized biomolecules.

FDLT powder was dissolved in sterile PBS at a concentration of 1 mg/mL and then filtered (Figure 1A). The soluble FDLT-derived biomolecules were analyzed by circular dichroism (CD), and the spectrum showed a double minimum (~210–220 nm) and a positive peak (~270 nm) (Figure 1B).

In particular, the analysis revealed a negative peak (~210 nm) and a prominent positive band (~267 nm), thus suggesting the presence of A-form RNA (Table 1) compared to other nucleic acid conformations and allowing us to distinguish A-form features consistent with RNA structures. Nevertheless, A-form signals may arise not only from single strands, but also from other RNA structures or even viral RNA contamination. However, such pathogen contamination is unlikely to occur, due to the FDLT pasteurization process at 70 °C, prior to lyophilization. In addition, FDLT was solubilized in sterile conditions and used for experiments. These data are supported by comparative literature [14] where B-form DNA typically exhibits CD bands at wavelengths slightly above 270 nm, while A-form RNA is characterized by a maximum around 265–270 nm. The absence of significant CD signals beyond 270 nm supports a low contribution from B-form DNA or other nucleic acid conformations. Moreover, CD revealed the absence of a characteristic negative band at ~245 nm, which is typically associated with B-DNA. In addition, we observed a double negative signal between ~210 and 225 nm, a region commonly attributed to proteins in α-helix conformation. This spectral pattern supports RNA as a predominant form, or at least a significant presence, over DNA components in the FDLT.

### 2.2. Effects of FDLT on Cell Viability

#### 2.2.1. Effects of FDLT on Porcine Fibroblast Cell Viability

**Figure 2:**
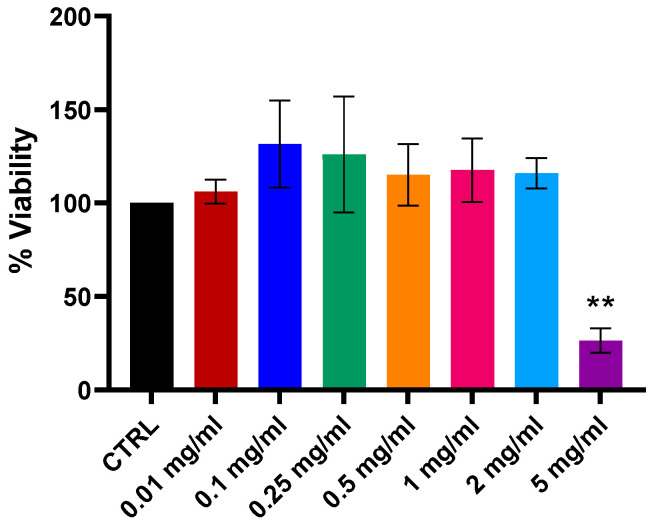
Cell viability of porcine fibroblasts treated with different concentrations of FDLT, showing no cytotoxicity at lower doses as measured by MTT assay. Statistical significance is indicated as follows: ** *p* < 0.01.

In a previous study, cytotoxicity of a lyophilized liver powder (LLP) was evaluated in normal canine adipose tissue-derived mesenchymal stromal cells (At-MSCs) [4]. The results showed that the treatment did not cause cytotoxicity in primary cell lines and was safe at concentrations from 0.01 to 2 mg/mL. For FDLT evaluation in canine At-MSCs, two different batches have been used (see Supplementary Results). To assess the cytotoxicity of FDLT on normal primary cell culture, dermal porcine fibroblasts were exposed to a concentration range of 0.01 to 5 mg/mL. MTT assay results demonstrated that cell viability remained unaffected up to 2 mg/mL compared to the serum-free control (Figure 2). A statistically significant reduction (*p* < 0.001) in cell viability was observed only at the highest concentration tested (5 mg/mL). Notably, the concentrations of 0.25 and 0.5 mg/mL, selected for experiments with the H1975 and PC9 lung cancer cell lines, did not induce cytotoxicity in primary porcine fibroblasts. We also observed the effect of FDLT treatment on cell migration by wound healing assay. FDLT combined with Osimertinib further reduced H1975 cell migration compared to Osimertinib alone (Supplementary Figure S1).

#### 2.2.2. Effects of FDLT on H1975 and PC9 Cell Viability

**Figure 3:**
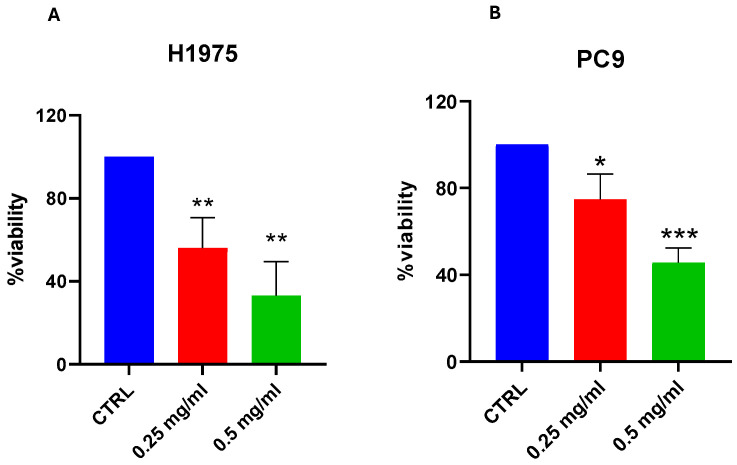
Effect of FDLT on cell viability. (**A**) MTS assay after 72 h incubation with FDLT 0.25 and 0.5 mg/mL showed dose-dependent reduction in H1975 cell viability. (**B**) MTS assay after 72 h treatment with FDLT 0.25 and 0.5 mg/mL resulted in a dose-dependent decrease in PC9 cell viability. Statistical significance is indicated as follows: * *p* < 0.05, ** *p* < 0.01, *** *p* < 0.001.

To determine the effect of FDLT on cell viability, a cell toxicity assay was performed using 0.25 mg/mL and 0.5 mg/mL FDLT for 72 h. The respective 0.25 mg/mL and 0.5 mg/mL doses of the FDLT were selected based on a dose response screening experiment, which proved that these doses produced a significant effect on lung cancer cells. Moreover, we observed that cell viability was reduced to 50% and 75% after exposure to 0.25 mg/mL and 0.5 mg/mL in the H1975 cell line, thus suggesting that FDLT can suppress cell viability in a dose-dependent manner (Figure 3A). Similar results were obtained for PC9 cells (Figure 3B). Considering that the highest concentration of FDLT was more effective in inducing cell toxicity, initial experiments were conducted using both 0.25 mg/mL and 0.5 mg/mL FDLT, but further experiments were performed using the highest dose at 0.5 mg/mL.

### 2.3. Cell Cycle Modulation in NSCLC Cells

**Figure 4:**
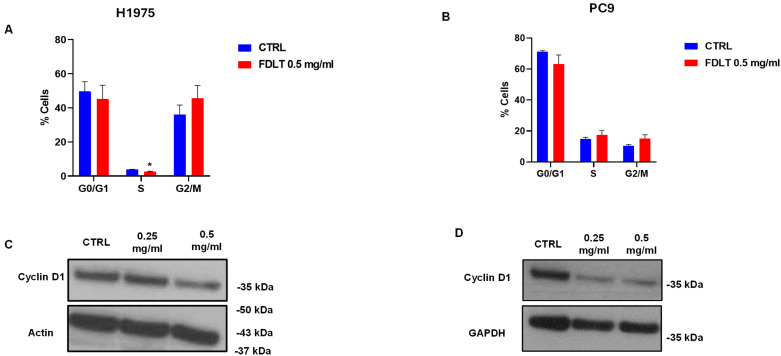
Cell cycle distribution and western blot analysis of cyclin D1. (**A**,**B**) The graphical representation of cell-cycle profiles for H1975 (**A**) and PC9 (**B**) cells demonstrated that FDLT treatment resulted in accumulation of cells in the G2/M phase and a corresponding reduction in progression to the G1 and S phases. (**C**,**D**) Western blot analysis of cyclin D1 levels in untreated and treated H1975 (**C**) and PC9 (**D**) cells showed that FDLT induced a marked decrease in protein expression. Data are presented as mean ± SD. Statistical significance is indicated as follows: * *p* < 0.05. GAPDH and Actin were used as loading controls.

To evaluate the effect of FDLT on cell cycle phases, H1975 and PC9 cells were treated with 0.5 mg/mL FDLT for 72 h, and the percentage of cells in the different phases was calculated. In particular, for the H1975 cell line exposed to FDLT, our results revealed an increase in the proportion of cells in the G2/M phase and a decrease in the proportion of cells in the G1 to S phase (*p* < 0.008) compared to untreated control, thus indicating that treatment caused cell cycle block in G2/M to G1 and S phase transition (Figure 4A). Similar results were obtained for PC9 cells (Figure 4B).

Cyclin D1 is a positive regulator of the cell cycle and drives G1 to S phase transition [15], and Western blot analysis was performed to evaluate its levels in whole cell lysates. In both NSCLC cell lines, protein expression of total cyclin D1 was reduced in a dose-dependent manner, thus indicating a reduction in cell proliferation and confirming cell cycle analysis (Figure 4C,D).

### 2.4. FDLT Suppresses Mitochondrial Membrane Potential

**Figure 5:**
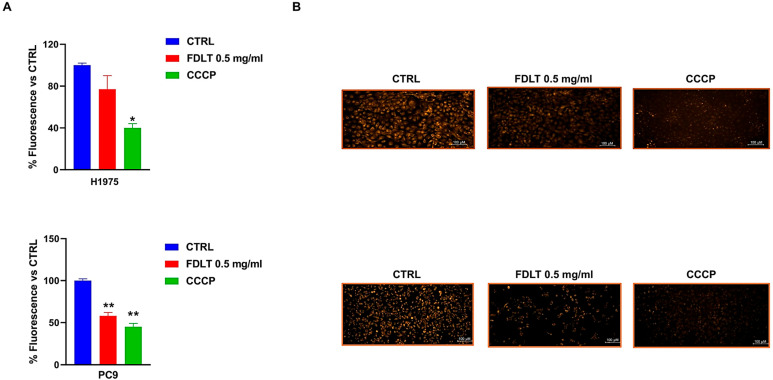
FDLT-dependent modulation of mitochondrial membrane potential. (**A**) Quantitative analysis of MMP modulation in both H1975 and PC9 cells treated with 0.5 mg/mL of FDLT after 72 h of incubation. Statistical significance is indicated as follows: * *p* < 0.05, ** *p* < 0.01; (**B**) Representative fluorescent images of MMP in H1975 and PC9 cells after 72 h of treatment. (Scale bar = 100 μm).

Effects of FDLT on modulation of mitochondrial membrane potential (MMP) were also assessed in NSCLC cells after 72 h of FDLT incubation. For this set of experiments, we used the TMRE probe, which is a cationic, red-orange, fluorescent dye that is sequestered by active mitochondria, whereas depolarized or inactive mitochondria fail to hold it. In particular, the untreated cells exhibited a higher fluorescent signal than treated ones, thus suggesting the occurrence of a FDLT-mediated mitochondrial depolarization (Figure 5A). CCCP was used as a positive control to validate loss of MMP. In summary, our findings suggested that FDLT in NSCLC cells may induce mitochondrial dysfunction, potentially leading to cell death through the activation of pro-apoptotic signaling pathways or increased oxidative stress. However, it is interesting to note that MMP loss can occur at earlier stages of the cell death process or as part of stress-induced signaling that does not immediately result in DNA fragmentation detectable as a sub-G1 peak during cell cycle analysis. Moreover, the most impressive effect was observed in PC9 cells. Representative images were captured with a high-resolution fluorescence microscope (20× magnification, Figure 5B).

### 2.5. FDLT Reduces EMT Transition in H1975 and PC9 Cells

**Figure 6:**
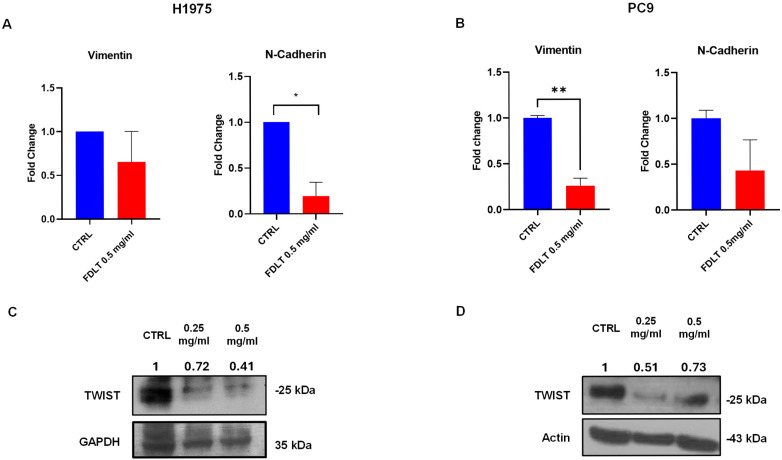
FDLT-dependent modulation of mRNA and proteins involved in EMT activation in NSCLC cells. (**A**,**B**) Vimentin and N-Cadherin mRNA levels were measured by qRT-PCR in (**A**) H1975 and (**B**) PC9 cells. Statistically significant difference as compared to CTRL * *p* < 0.05, ** *p* < 0.01). (**C**,**D**) Western blot analysis determined whole cell lysates of Twist1 protein expression levels in (**C**) H1975 and (**D**) PC9 cells. Quantitative analysis of protein expression was also performed. β-actin was considered as a loading control.

Vimentin and N-cadherin are crucial markers of EMT transition in cancer cells [16] [17], and here we found a significant reduction in mRNA levels of Vimentin and N-Cadherin in both FDLT-treated NSCLC cell lines (Figure 6A,B). In particular, for H1975 cells, treatment induced a significant reduction in N-Cadherin, whereas for PC9 cells, an impressive decrease in Vimentin was observed.

Furthermore, protein expression of EMT transcription factor (TF) Twist1 was also analyzed after incubating cells for 72 h with FDLT. Twist1 significantly promotes the EMT pathway and cancer cell invasion, hence favoring cancer metastasis [18]. Through immunoblotting, we observed a significant reduction in Twist1 levels in FDLT-treated cells as compared to control, and the results were confirmed by quantitative analysis of protein bands in both cell lines (Figure 6C,D).

### 2.6. Effect of FDLT Treatment on Colony Formation

**Figure 7:**
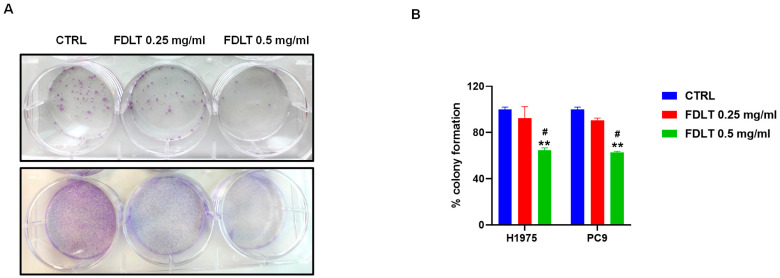
(**A**) Colony formation in untreated and treated NSCLC cells. (**B**) FDLT treatment led to a significant reduction in cell colony formation in both cell lines. Statistical significance is indicated as follows: # *p* < 0.05, versus FDLT 0.25 mg/ml; ** *p* < 0.01. versus CTRL.

To evaluate the long-term effect of FDLT treatment on cell growth and proliferation of NSCLCs, the ability of a single cell to survive and proliferate into a colony was monitored after 10–15 days of treatment. We found that FDLT stimulation was able to decrease, in a dose-dependent manner, the colony numbers in both H1975 and PC9 cell lines (Figure 7A). Quantitative analysis was also performed by colorimetric assay, and we found a significant reduction in cell growth at a concentration of 0.5 mg/mL FDLT (Figure 7B). These findings support our hypothesis about the role of soluble FDLT-derived biomolecules in promoting cell growth arrest and inhibiting proliferation, thus in agreement with cell cycle modulation and cyclin D1 expression.

## 3. Discussion

In recent years, several efforts have been made to develop new therapeutic strategies to avoid resistance and improve tumor response. It is well known that the most aggressive tumors are associated with uncontrolled proliferation, increased cell migration, and high levels of EMT markers [19]. We conducted an exploratory study to evaluate the biological effect of bioactive molecules derived from FDLT on cell growth and EMT activation. In particular, pigs may be considered a good preclinical model to investigate new therapies for human diseases due to their similarities to humans in anatomy, physiology, and immune responses [20]. It is also interesting to note that in each organ, the presence of mesenchymal stem cells (MSCs) may contribute to the composition of biological factors released from each organ in the body (secretome), and specifically, lung-derived MSCs may be an effective tool for the treatment of lung disorders [21].

In the present study, we showed that the soluble bioactive molecules derived from pig FDLT suspension are enriched in nucleic acids and proteins and may be a useful tool for lung cancer treatment.

Preliminary analyses were conducted on two types of primary cell cultures to assess the in vitro safety profile of FDLT. The two types of primary cell cultures used in the study were porcine dermal fibroblasts and canine adipose tissue derived-mesenchymal stromal cells (At-MSCs). At-MSCs were selected as a model due to their previous use in a safety analysis of a porcine lyophilized liver powder (LLP) that was prepared using a procedure similar to that of FDLT [4]. In porcine fibroblast cultures, FDLT demonstrated toxicity only at 5 mg/mL concentration. No signs of toxicity were observed at concentrations of 0.25 and 0.5 mg/mL, which were employed in the lung cancer cell studies conducted in this work. Cytotoxicity analyses on canine MSCs (see Supplementary Results, Figure S2 and [22]) further supported the safety of FDLT. Notably, concentrations of up to 2 mg/mL resulted in increased cell viability compared to the serum-free control, while a cytotoxic effect was only observed at a concentration of 5 mg/mL.

Having established a favorable safety profile in non-malignant primary cells, we next investigated the specific anti-tumor mechanisms in non-small cell lung cancer (NSCLC).

In summary, we demonstrated that biological factors contained in our FDLT sample may modulate gene transcription and levels of proteins linked to cell growth, cell cycle, and the EMT program. Interestingly, we found that FDLT treatment was able to reduce cell viability, depolarize the mitochondrial membrane, and cause cell cycle arrest in two NSCLC cell lines. From the MMP assay, we observed loss of membrane potential in both H1975 and PC9 cells treated with 0.5 mg/mL FDLT, thus likely leading to apoptosis and cell death. These findings correlate with research carried out by other research groups [23] that demonstrated the effect of soluble factors secreted by mesenchymal cells and involved in inhibitory mechanisms of NSCLC cell duplication and through paracrine mechanisms [24]. In agreement with this concept, we also investigated the effect of FDLT on cell proliferation by performing cell cycle analysis and a clonogenic assay. In particular, we found that FDLT was able to reduce the cell numbers in the S phase of the cell cycle, thus causing cell growth arrest at G2/M phase and a reduction in cell colony formation. We speculate that such results may be linked to the evidence that different cell types in the body can secrete biological factors involved in tissue homeostasis and able to modulate the proliferation states of each organ [24].

In accordance with these findings, we also demonstrated that FDLT stimulation may be helpful to inhibit or prevent the occurrence of EMT in NSCLC cells, thus hindering the acquisition of the most aggressive phenotype. Vimentin and N-cadherin, as well as Twist reduction, have been identified as useful biomarkers of tumor response to FDLT stimulation. From a biomolecular constitutive perspective, the extract of FDLT was found to contain a significant proportion of RNA among its nucleic acid components, as evidenced by CD spectroscopic analysis of the characteristic bands observed in our experiments (Figure 1). This aspect suggested to us that possible microRNA (miRNA) may be contained in the sample and modulate the expression of cyclin D1 and EMT markers. But this hypothesis requires verification through subsequent miRNA omics analysis and loss-of-function experiments. Similarly, the protein components, including transcription factors, may affect the expression of genes involved in cancer cell growth and proliferation as well as EMT activation. Future experiments will be necessary to better characterize FDLT and shed light on the molecules able to inhibit NSCLC progression; also, the effect of FDLT on immune cells should be explored to better understand the expected impact of FDLT on certain pathways involved in maintenance and regeneration. However, although in the present study, we did not elucidate the exact molecular composition of FDLT, our results showed a relevant impact for translational medicine and for preparing the development of new therapies to inhibit the proliferation of resistant clones.

## 4. Materials and Methods

### 4.1. Preparation of Porcine Lung Extract

Lyophilized lung porcine extracts were received from Neorland s.r.l. (Cremona, Italy). Young Italian-origin healthy male and female pigs (consisting of 150 kg in weight) were slaughtered, and soon after slaughtering, the organs were isolated carefully. The slaughtering process was performed in accordance with the EU/regulations 852/04 and 853/04, and animal welfare requirements were also met as described in the EU rules 2073/05 and 1099/09. After isolation, the organs were packed and transported to the freezing phase (−20 °C). The organs were subjected to sublimation, followed by a drying process in which all the water was removed. The various steps of the freeze-drying procedure were followed according to the guidelines mentioned by producers (involving various temperature-lowering steps). The organs were then ground, and after removal of impurities, were packed in a triple-layer aluminum bag [4,25]. The same method was used for the preparation of porcine lung extract. Two different FDLT batches (A and B) (see Supplementary Materials) were tested in the analyses of normal primary cell lines. Batch B was employed for the analyses of the NSCLC cell lines.

### 4.2. Antibodies and Reagents

For immunoblotting, the following antibodies were used: Cyclin D1 (rabbit polyclonal; 1:500; Cell Signaling Technology Cat# 2922, RRID: AB_2228523), TWIST 1 (rabbit polyclonal, 1:1000; Sigma-Aldrich Cat# T6451, RRID: AB_609890), GAPDH (2118, Cell Signaling Technology, Danvers, MA, USA), beta Actin (SAB1305554, Sigma-Aldrich, St. Louis, MO, USA). The porcine lyophilized lung tissues were provided by Dr. Orlando Ferroni (Neorland^®^, Cremona, Italy).

### 4.3. Solubility of FDLT Powder

FDLT powder was solubilized by dissolving 1 mg/1 mL of the FDLT powder in sterile PBS or SF RPMI medium (Gibco, Grand Island, NY, USA). The dissolved powder was then stirred on a stirrer for 1 h and then filtered inside a biosafety cabinet with syringe filters, and was ready to be used for the required treatment.

### 4.4. Cell Culture

#### 4.4.1. Isolation and Culture of Porcine Fibroblasts

Porcine skin biopsies were collected from discarded tissue at a local slaughterhouse. Fibroblast isolation was performed according to Zou et al. [26] with slight modifications. Tissue samples were washed in 70% ethanol and PBS (pH 7.4), followed by dispase II digestion (2 U/mL, 2 h at 37 °C) in DMEM to remove the epidermis. The dermis was then minced in 1–2 mm^3^ fragments and digested with 0.1% (*w*/*v*) collagenase type I in DMEM. The resulting single-cell suspension was centrifuged (150× *g*, 15 min) and resuspended in DMEM supplemented with 10% FBS and antibiotics (100 μg/mL penicillin/streptomycin, 2.5 μg/mL amphotericin B). Cells were cultured at 37 °C under 5% CO_2_, passaged using 0.05% Trypsin-EDTA upon reaching sub-confluence, and reseeded at 5000 cells/cm^2^. Cells at passages 3–4 were used for experiments.

#### 4.4.2. Culture of Tumor Cell Lines

Non-small cell lung cancer cells bearing mutant EGFR (H1975) and PC9 cell lines were purchased and authenticated by the American Type Culture Collection (ATCC, Manassas, VA, USA). Briefly, as previously described by De Rosa [27], both cell lines were grown in RPMI 1640 (Gibco, Grand Island, NY, USA) medium supplemented with 10% fetal bovine serum (FBS), 100 IU/mL penicillin, and 50 µg/mL streptomycin in a humidified incubator in 5% CO_2_ at 37 °C. Different concentrations of FDLT powder (0.25 mg/mL, 0.5 mg/mL) were used to evaluate in vitro cytotoxicity on NSCLC-H1975 and PC9 cells.

### 4.5. CD Spectroscopy

To evaluate the properties of DNA, RNA, miRNA, and proteins in our FDLT sample, an initial spectroscopic analysis was performed to better understand the function of bioactive molecules present in FDLT. The FDLT sample was reconstituted in sterile phosphate-buffered saline (PBS, pH 7.4) at a concentration of 1 mg/mL and incubated under shaking for 1 h at room temperature (RT, ~25 °C) to allow homogenous dispersion of the particles. The suspension was subsequently filtered through a 0.2 µm sterile filter to remove any particulate contaminants. CD and UV spectra were acquired using a JASCO J-715 spectropolarimeter (Tokyo, Japan) equipped with a Peltier-controlled cell holder. Measurements were performed using a 1 cm pathlength quartz cuvette, with spectral acquisition in the range of 320–204 nm, at 1 nm data pitch and bandwidth of 1.0 nm, with a response time of 1 s and a scanning speed of 100 nm/min. The CD signal of the buffer alone was recorded under identical conditions and subtracted from the FDLT bio-molecular mixture spectrum to isolate the contribution of the bioactive components. All spectra were acquired at 25 °C [28].

### 4.6. Cell Cytotoxicity and Proliferation Assay

FDLT-induced cytotoxicity was assessed through MTS assay (Cell Counting Kit-8, Dojindo Laboratories, Kumamoto, Japan) in NSCLC H1975 and PC9 cells. Briefly, cells were seeded in a 96-well plate at a density of 1 × 10^4^ cells/well in 100 µL of complete RPMI medium. After 24 h, cells were treated with FDLT at 0.25 mg/mL and 0.5 mg/mL doses. Later, the 0.5 mg/mL concentration of FDLT was employed in further experiments, as it gave notable results. Viable cells were determined spectrophotometrically at 450 nm and expressed as the cell percentage, considering the untreated control as 100%.

In porcine fibroblast cells, FDLT cytotoxicity was assessed via MTT assay. Porcine fibroblasts were plated in 96-well plates (5 × 10^3^ cells/well) and allowed to adhere for 24 h. Cells were then exposed to FDLT (0.01–5 mg/mL in serum-free medium) or serum-free medium (control) for 48 h. Six replicates were used for each condition across four independent experiments (*n* = 4). Absorbance of the converted formazan dye was measured at 570 nm (reference: 630 nm). Metabolic activity was expressed as the percentage of untreated control. FDLT cytotoxicity in canine At-MSCs using two different batches is shown in Supplementary Figure S2.

### 4.7. Mitochondrial Membrane Potential MMP Assay

For the MMP assay, 2 × 10^4^ cells were seeded in a 96-well plate, and after 24 h, the cells were treated with 0.5 mg/mL FDLT. After 72 h, a positive control for MMP loss, CCCP, was added at a final concentration of 50 µM in the positive control wells and incubated at 37 °C for 5 min. Then, 2 µM of TMRE staining solution was added to each well, and the plate was placed in an incubator for 20 min. The plate was then washed with PBS, and fluorescence intensity was measured at Ex/Em: 550/580 nm with a fluorescence microplate reader (VICTOR^®^ Nivo™ multimode plate reade, Revvity Inc., Singapore). In addition, images were recorded with a fluorescence microscope (ECLIPSE Ti2, Nikon, Tokyo, Japan).

### 4.8. Immunoblotting

Western blot of whole cell lysates was performed using a standard procedure.

Samples were resolved by SDS-PAGE gels and transferred onto PVDF membranes (Millipore, Burlington, MA, USA). The membrane was blocked with 5% milk, and the primary antibodies were incubated overnight. Horseradish peroxidase-linked anti-rabbit (BioRad Laboratories, Hercules, CA, USA) and anti-mouse (BioRad) antibodies were used as secondary antibodies. A commercially available ECL kit (Advansta, San Jose, CA, USA) was used to reveal protein bands that were quantified by morpho-densitometric analysis using ImageJ software version Java8 (NIH, Bethesda, MD, USA). As loading controls, GAPDH and Actin were both employed to improve the resolution of protein bands.

### 4.9. Gene Expression Analysis by qRT-PCR

Trizol reagent was added to the cell pellets and centrifuged at 12,000 rpm for 10 min. Then, 100 μL of chloroform was added to the samples, and the tubes were shaken to facilitate phase separation, after which centrifugation was carried out at 15,000 rpm for 15 min. The aqueous phase containing RNA was transferred to new tubes, and isopropanol was added to precipitate RNA. Samples were centrifuged again at 15,000 rpm for 15 min. Supernatant was discarded, and the pellets were washed with 70% ethanol. The washing step was carried out multiple times. After the final washing step, the supernatant was carefully discarded, and the pellets were allowed to air dry in a chemical hood for 30 min and then resuspended in RNAse-free water. RNA concentration and its purity were assessed by the Nanodrop 2000 spectrophotometer (Thermo Fisher Scientific, Waltham, MA, USA), by measuring absorbance at 260/280 nm. For cDNA preparation, 1 μg RNA from each sample was used for reverse transcription using the SensiFAST cDNA Synthesis Kit (BIO-65053, Meridian Bioscience, Memphis, TN, USA). The reverse transcription method included a first step incubation at 25 °C for 10 min, followed by 42 °C for 15 min, and a final step at 85 °C for 5 min.

To analyze the effect of EMT genes in FDLT-treated samples, qRT-PCR analysis was performed. Primer information is listed in Table S1 of the Supplementary Materials. mRNA expression levels of vimentin and N-cadherin were assessed by using a QuantStudio 7-Flex instrument (Applied Biosystems by Life Technologies, Monza, Italy) and the Sensi FAST SYBR Hi-ROX Kit (BIO-92005, Meridian Bioscience, Memphis, TN, USA). The relative expression of genes was determined by normalization to 18S, serving as an internal control gene. Relative gene expression values were measured using 2^−ΔCT^ and 2^−ΔΔCT^. Normalization of mRNA was performed using 18S as a reference control. Data are presented as Mean ± SD.

### 4.10. Clonogenic Assay

A single cell suspension of 800 cells per well was seeded in a 6-well plate (for H1975 and PC9). The next day, cells were treated with 0.25 and 0.5 mg/mL of FDLT, and the medium was refreshed every 2–3 days. After 15 days, cells were fixed with 70% ethanol and stained with 1% crystal violet. When the plates were completely dried, colonies were read on the plate, dissolved, and the OD of crystal violet was measured at 570 nm with the VICTOR^®^ Nivo™ multimode plate reader.

### 4.11. Cell Cycle Distribution Analysis

For cell cycle analysis, 1 × 10^5^ cells were plated. When they reached 40% confluency, the cells (H1975 and PC9) were treated with 0.5 mg/mL FDLT and kept in the incubator for 72 h at 37 °C. After 72 h incubation, the cells were collected, counted by hemocytometer, and resuspended in ice-cold 96% ethanol. Cells were then vortexed and fixed at 4 °C for 30 min. After fixation, the cells were centrifuged at 300× *g* for 5 min. Next, 200 μL of Propidium iodide (50 μg/mL, Sigma-Aldrich) and RNasi (1 mg/mL, Sigma-Aldrich) was added, and the cells were kept in the dark for 30 min. The cells were analyzed with BD FACS ARIA III (Becton & Dickinson, Mountain View, CA, USA), BD FACSDiva™8.0 Software (Becton & Dickinson, Mountain View, CA, USA). The experiment was performed at least in duplicate.

### 4.12. Statistical Analysis

Experimental data are presented as Mean ± SD. Statistical differences were calculated with (one-way and multiple comparison) ANOVA and *t*-test using (GraphPad Prism software, version 8). A *p*-value < 0.05 was considered statistically significant.

## 5. Conclusions

FDLT-derived bioactive molecules from healthy lungs may include biological factors that can have a variety of significant therapeutic effects. These comprise an increase in cancer cell growth arrest and death, as well as a decrease in the expression of EMT gene. From a molecular composition standpoint, CD spectroscopic analysis of the characteristic bands revealed in our experiments indicated that the FDLT extract may contain proteins and a substantial amount of RNA within its nucleic acid profile. Hence, future efforts in RNA sequencing will be required for unambiguous characterization and validation of the bioactive factors present in the analyzed samples. In summary, the current study indicated that freeze-dried lyophilized powder derived from porcine lung is safe to use, even if additional efforts are necessary to develop new therapeutic approaches for lung treatment with the aim of inhibiting cancer cell proliferation and migration. In particular, further studies should be performed in the field to exactly identify the factors and cellular pathways that drive cell growth arrest and reversal of EMT phenotype upon FDLT stimulation.

## Figures and Tables

**Table 1:** Characteristic circular dichroism bands for B-DNA and A-RNA.

Spectral Feature	B-DNA	A-RNA
Positive band (nm)	~275–280	~265–270
Negative band (nm)	~245	~210–220
Helical conformation	Right-handed B-form helix	Right-handed A-form helix

## Data Availability

The original contributions presented in this study are included in the article/Supplementary Materials. Further inquiries can be directed to the corresponding author.

## Supplementary Materials

The following supporting information can be downloaded at: https://www.mdpi.com/article/10.3390/ijms262311743/s1.

## Abbreviations

FDLT	Freeze dried lung tissue
EMT	Epithelial mesenchymal transition
NSCLC	non-small cell lung cancer cell line
MMP	Mitochondrial membrane potential
MSCs	Mesenchymal stem cells
EVs	Extracellular vesicles
PBS	Phosphate buffer saline
CD	Circular dichroism
FBS	Fetal bovine serum
CCCP	Carbonyl cyanide m-chloro phenyl hydrazone
FACS	Fluorescence activated cell sorting
SF	Serum-free
SD	Standard deviation
