# Supplementary material for: Effect of Soluble Factors Released from Porcine Freeze-Dried Lung Tissue (FDLT) on Modulation of Cell Growth and EMT Signature in Non-Small Cell Lung Cancer (NSCLC)—A Preliminary In Vitro Study
Source: Int J Mol Sci. 2025 Dec 4;26(23):11743. doi: 10.3390/ijms262311743 (PMC12692275; doi:10.3390/ijms262311743)
Supplement: Supplementary file 1 [file ijms-26-11743-s001.zip › ijms-3945277-supplementary.pdf]

Supplementary Data

Table S1. List of Primer sequences used for qRT-PCR.

Gene	Forward primer sequence	Reverse primer sequence
18s	5'-CGCCGCTAGAGGTGAAATTC-3′	3' -CTTTCGCTCTGGTCCGTCTT-5′
Vimentin	5'-AGGCAAAGCAGGAGTCCACTGA-3′	3′-ATCTGGCGTTCCAGGGACTCAT-5'
N-cadherin	5'-CCTCCAGAGTTTACTGCCATGAC-3'	3′-GTAGGATCICCGCCACTGATTC-5'

Wound healing assay

To evaluate the potential synergistic effect of FDLT with standard drug, Osimertinib on H1975 cell migration, we performed a wound healing experiment comparing Osimertinib alone (0.5uM) and in combination with FDLT. Our results indicate that treatment with FDLT in combination with Osimertinib further suppressed the migration ability of H1975 cells compared to treatment with Osimertinib alone, suggesting a potential effect on decreasing cell migration (as shown in figure S1 below). Thus, we hypothesize that FDLT may provide a beneficial modulating effect, that deserve further studies.

Wound healing analysis of H1975, one of the two cell lines.

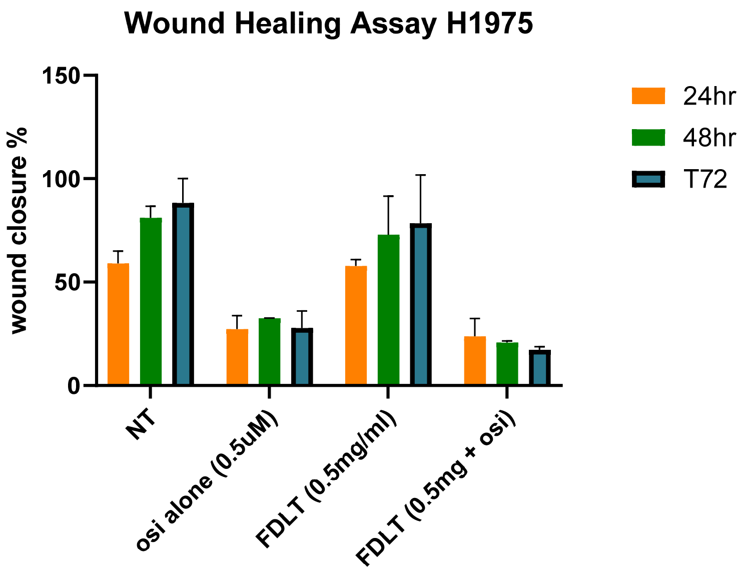

Figure S1. Wound healing assay of H1975 shows FDLT + OSI treatment suppressed the migration ability of H1975 cells, suggesting a potential synergistic effect on inhibiting cell migration.

Effects of FDLT on canine adipose tissue-derived Mesenchymal Stromal Cells (At-MSCs) viability

Canine mesenchymal stromal cells were used to evaluate the cytotoxicity of FDLT on normal primary cells. The same cells were previously used to evaluate the safety of a lyophilized porcine liver powder (4). Two different FDLT batches were used and tested in populations of At-MSCs derived from six donors, thereby limiting the donor effect. The assessment of the safety of in vitro use of FDLT was a preliminary step to the analysis of its effects on NSCLC cells.

Results

Effects of FDLT on viability of canine cells. A dose-response curve was observed after treating primary cultures of At-MSCs with FDLT concentrations ranging from 0.1 to 5 mg/ml (Figure S2). All tested concentrations resulted in significantly higher viability compared to the serum-free control ($p < 0.001$), reaching a plateau at 2 mg/ml. Although FDLT batch B (Figure S2, B) showed a slight decrease in cell viability at 5 mg/ml, no toxicity was observed relative to the serum-free control. These results indicate that FDLT lacks cytotoxic effects up to 5 mg/ml, highlighting the *in-vitro* safety of the preparation.

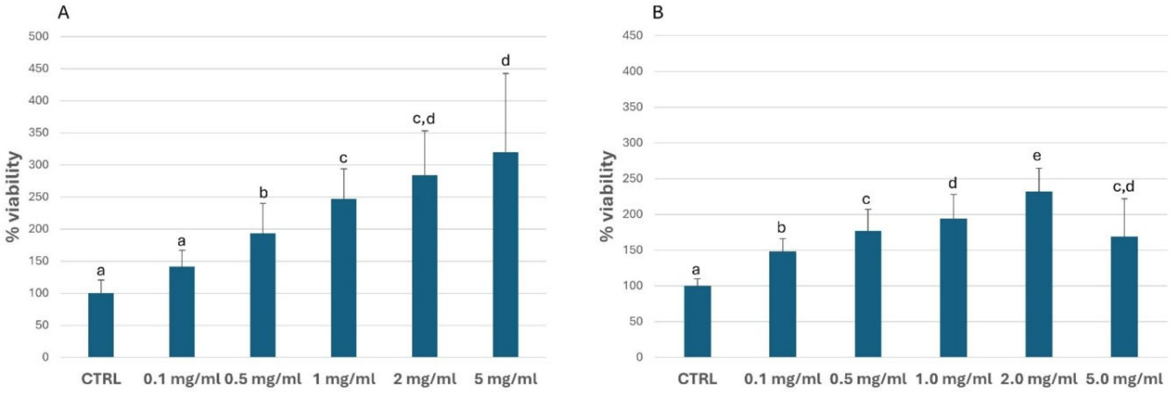

Figure S2. Effects of two different batches (A, B) of FDLT on At-MSCs viability. MTT assays were performed with 6 different cell populations for each batch, at concentrations ranging from 0.1 to 5 mg/ml. No cytotoxic effect was observed for either batch. Viability is expressed as a percentage relative to the serum-free control. Absence of a common letter indicates a statistically significant difference ($p < 0.001$).
